# Health status and health systems financing in the MENA region: roadmap to universal health coverage

**DOI:** 10.1186/s41256-017-0044-9

**Published:** 2017-09-04

**Authors:** Eyob Zere Asbu, Maysoun Dimachkie Masri, Amer Kaissi

**Affiliations:** 1 Health System Financing Division, Health Authority Abu Dhabi, Abu Dhabi, United Arab Emirates; 2 Department of Healthcare Administration, Trinity University, San Antonio, TX USA

**Keywords:** Millennium development Goals (MDGs), Sustainable development Goals (SDGs), Universal health coverage (UHC), Middle East and North Africa (MENA), Health system financing, Out-of-pocket spending (OOPS), Health status

## Abstract

**Background:**

Since the declaration of the Millennium Development Goals (MDGs) in 1990, many countries of the Middle East and North Africa (MENA) region made some improvements in maternal and child health and in tackling communicable diseases. The transition to the global agenda of Sustainable Development Goals brings new opportunities for countries to move forward toward achieving progress for better health, well-being, and universal health coverage. This study provides a profile of health status and health financing approaches in the MENA region and their implications on universal health coverage.

**Methods:**

Time-series data on socioeconomics, health expenditures, and health outcomes were extracted from databases and reports of the World Health Organization, the World Bank and the United Nations Development Program and analyzed using Stata 12 statistical software. Countries were grouped according to the World Bank income categories. Descriptive statistics, tables and charts were used to analyze temporal changes and compare the key variables with global averages.

**Results:**

Non-communicable diseases (NCDs) and injuries account for more than three quarters of the disability-adjusted life years in all but two lower middle-income countries (Sudan and Yemen). Prevalence of risk factors (raised blood glucose, raised blood pressure, obesity and smoking) is higher than global averages and counterparts by income group. Total health expenditure (THE) per capita in most of the countries falls short of global averages for countries under similar income category. Furthermore, growth rate of THE per capita has not kept pace with the growth rate of GDP per capita. Out-of-pocket spending (OOPS) in all but the high-income countries in the group exceeds the threshold for catastrophic spending implying that there is a high risk of households getting poorer as a result of paying for health care.

**Conclusion:**

The alarmingly high prevalence of NCDs and injuries and associated risk factors, health spending falling short of the GDP and GDP growth rate, and high OOPS pose serious challenges for universal health coverage. Using multi-sector interventions, countries should develop and implement evidence-informed health system financing roadmaps to address these obstacles and move forward toward universal health coverage.

## Background

On September 25, 2015, the United Nations General Assembly adopted a new development agenda, ‘Transforming our World: The 2030 Agenda for Sustainable Development’ [1]. The new agenda, which builds on the Millennium Development Goals (MDGs) of 1990 consists of 17 Sustainable Development Goals (SDG) and 67 indicators to measure progress set for 2030 [2, 3].

Health is centrally placed within the new agenda. First, SDG 3 aims to ‘Ensure healthy lives and promote well-being for all ages’ and its 13 targets cover all major health topics, including reproductive, maternal, new born and child health, infectious diseases, non-communicable diseases (NCD), mental health, and road traffic injuries. Second, almost all of the other 16 SDGs are either directly related to health or indirectly contribute to health [3, 4].

Universal Health Coverage (UHC) is central to SDG 3 and its linkages with the other SDGs. UHC not only supports all other targets but also is key to the implementation of the targets [3]. UHC started to gain attention in 2005, when the World Health Assembly recognized the importance of the role of State Legislative and executive bodies in reforming health-financing systems to achieve universal coverage [5]. In order to reform healthcare financing systems, policymakers were urged to: first, adopt the risk pooling method and the prepayment approach for healthcare financial contributions as a way for increasing population coverage; second, to ensure adequate and equitable distribution of good quality healthcare systems; and third, to ensure sustainable funding of health programs or activities [5]. Later, the 2008 World Health Report on Primary Healthcare [6], re-emphasized the importance of UHC in creating or building health equity through financial protection and reduction in catastrophic expenditure or out-of pocket payment. This was followed by the 2010 World Health Report on Health Financing for UHC [7], which presented ways and options for raising sufficient resources and removing financial barriers to healthcare access. According to the latter report UHC means that all people can use the promotive, preventive, curative, rehabilitative and palliative health services they need, of sufficient quality to be effective, while also ensuring that the use of these services does not expose the user to financial hardship [7]. Achieving UHC requires the existence of a legal mandate and political commitment for universal access to healthcare services [8]. Furthermore, other socio-economic drivers may either facilitate or hinder UHC system development. Countries differ in their social, economic and political drivers that will contribute toward achieving and maintaining UHC [9].

Since the advent of the MDGs, the Middle East and North Africa (MENA) region made improvements in reducing child mortality, improving maternal health, and tackling communicable diseases [4, 10]. Despite those improvements, some countries of the region are currently in turmoil with Syria, Iraq, Libya and Yemen in war. The negative consequences of the wars including the spillover effects of the refugee crisis on health and health systems of the specific countries and the entire region cannot be overemphasized [11].

In an effort to improve health outcomes in the MENA region and achieve universal health coverage, different countries have to overcome different sets of challenges including the war effect [9, 12]. One of the challenges faced is how health should be financed so that all people are able to receive needed health services of sufficient quality without being exposed to financial hardship as a result of using the services [7, 9, 13]. This exploratory study provides a profile of health status and health financing approaches in the MENA region and their implications on UHC. The study reviews health care financing indicators in the MENA region as it relates to three main functions: revenue collection or raising, pooling of funds, and purchasing [13–15]. It attempts to highlight policy changes and recommends health-financing system reforms to move toward UHC and achieve better healthcare outcomes in the MENA region.

This descriptive study is expected to contribute to policy debates and generating hypotheses for future analytical and in-depth studies on specific issues related to burden of diseases and health systems building blocks, particularly health systems financing in the region and the countries covered in the context of universal health coverage.

## Methods

The following 17 countries of the MENA region are included in this study based on availability and comparability of data: Algeria, Bahrain, Egypt, Iraq, Jordan, Kuwait, Lebanon, Libya, Morocco, Oman, Qatar, Saudi Arabia, Sudan, Syria, Tunisia, the United Arab Emirates and Yemen. In this study, the following have been excluded from the MENA region of the World Bank: Djibouti, Iran, Israel and the West Bank and Gaza. These were excluded due to differences in social context, and in some cases non-availability of comparable data specifically in the required variables.

In this study, the various countries are grouped according to the World Bank income categories. Low middle income countries (LMICs) group includes Egypt, Morocco, Sudan, Syria, Yemen; Upper middle income countries (UMICs) group includes Algeria, Iraq, Jordan, Lebanon, Libya, and Tunisia; and, the high income counties (HICs) group consists of Bahrain, Kuwait, Oman, Qatar, Saudi Arabia, and the United Arab Emirates.

Data were mainly extracted from databases and reports of the World Health Organization and other United Nations agencies. Time series data on health financing indicators for the period 2000 to 2014 was extracted from the WHO’s global health expenditure database [16]. The indicators included are: (i) per capita total expenditure on health; (ii) total expenditure on health as a share of GDP; (iii) general government expenditure on health as a share of GDP; (iv) General government expenditure on health as a share of general government expenditure; (v) general government expenditure on health as a share of total expenditure on health; and (vi) out-of-pocket expenditure as a share of total expenditure on health.

Data on estimated disability-adjusted life years (DALYs) by cause for major burden of disease categories for the year 2012 was extracted from the WHO’s 2014 estimates of DALYs [17]. The broad categories included were: (i) infectious and parasitic diseases; (ii) respiratory infections; (iii) maternal conditions; (iv) nonatal conditions; (v) nutritional deficiencies; (vi) non-communicable diseases; and (vii) injuries. For a closer look on non-communicable diseases, which are highly prevalent in most of the countries covered in this study, data was also extracted on the following conditions: diabetes mellitus, cardiovascular diseases, mental and behavioral disorders and malignant neoplasms.

Risk factors, which include prevalence of raised fasting blood glucose, raised blood pressure and obesity rate in the population 18 years and above; and prevalence of tobacco smoking in the population 18 years and above was obtained from the World Health Statistics 2015 [18]. The same source was also used to extract data on life expectancy, under-five mortality rate, maternal mortality ratio and health workforce densities. Human development index data was extracted from the Human Development Report 2015 [19].

Descriptive statistics, percentages and graphs were computed using MS Excel and Stata 12 statistical software. Country statistics were compared within the group and against global averages disaggregated by World Bank income status.

## Results

### Socioeconomic characteristics

The MENA countries included in this study are a diverse group of countries comprising high, upper-middle and lower-middle-income countries. Table 1 depicts selected health and development indicators of the study countries. In 2014, the mean per capita Gross National Income (GNI) (in 2011 PPP $) ranged from as low as US$ 5484 for the LMICs to as high as US$ 65,705 for the HICs (Table 1). Two of the LMICs (Sudan and Yemen) had Human Development Indices (HDI) less than 0.550, and are classified as low human development countries. In contrast, all of the HICs except Oman are classified as very high human development countries (HDI ≥ 0.8000) (Table 1). The HDI reflects the ability of a country to achieve long and healthy life, the people being knowledgeable and have a decent standard of living. The GNI per capita rank minus the HDI rank is negative in an overwhelming majority of the countries (14/17) indicating that they are ranked better in GNI per capita than in HDI and implying that there is room to improve their performance in human development. Adult literacy rate in the region ranges from 66% in Yemen to 98% in Jordan. Most of the countries (10/17) have a level of adult literacy that is higher than the global average of 84%.

**Table 1:** Selected Socioeconomic and Health Indicators for the Middle East and North Africa Region

Indicator	Algeria	Bahrain	Egypt	Iraq	Jordan	Kuwait	Lebanon	Libya	Morocco	Oman	Qatar	Saudi Arabia	Sudan	Syrian Arab Republic	Tunisia	United Arab Emirates	Yemen
Gross National Income per capita 2014 (at 2011 PPP$)*	13,054	38,599	10,512	14,003	11,365	83,961	16,509	14,911	6850	34,858	123,124	52,821	3809	2728	10,404	60,868	3519
Human Development Index 2014	0.736	0.824	0.690	0.654	0.748	0.816	0.769	0.724	0.628	0.793	0.850	0.837	0.479	0.594	0.721	0.835	0.498
Adult literacy rate (%) (15+ years) (2005–2013)	72.6	94.6	73.9	79.0	97.9	95.5	89.6	89.9	67.1	86.9	96.7	92.4	73.4	85.1	79.7	90.0	66.4
Total population, 2013 (‘000)	39,208	1332	82,056	33,765	7274	3369	4822	6202	33,008	3632	2169	28,829	37,964	21,898	10,997	9346	24,407
Population 60+ (2013) (%)	7	3	9	5	5	4	12	7	8	4	2	5	5	6	11	1	5
Life expectancy at birth (2013) (years)	72	77	71	70	74	78	80	75	71	76	79	76	63	76	76	77	64
Healthy life expectancy at birth (2013) (years)	62	66	62	61	64	68	70	64	61	66	68	65	53	66	66	67	55
Infant mortality rate per 1000 live birth	21.6	5.2	18.6	28.0	16.0	8.1	7.8	12.4	26.1	9.8	7.0	13.4	51.2	11.9	13.1	7.0	40.4
Under 5 mortality rate per 1000 live birth	25	6	22	34	19	10	9	15	30	11	8	16	77	15	15	8	51
Maternal mortality ratio per 100,000 live births	89	22	45	67	50	14	16	15	120	11	6	16	360	49	46	8	270
Physician per 10,000 population, 2007–2013	12.1	9.2	28.3	6.1	25.6	17.9	32.0	19.0	6.2	24.3	77.4	24.9	2.8	14.6	12.2	25.3	2.0
Nursing and midwifery personnel per 10,000 population, 2007–2013	19.5	23.7	35.2	-	40.5	45.5	27.2	68.0	8.9	53.8	118.7	48.7	8.4	18.7	32.8	31.6	6.8

Sources of data: [18, 19]

*PPP* purchasing power parity

The countries in the group also vary in terms of their population size, ranging from a population of less than 1.5 million (Bahrain) to a high of over 82 million (Egypt). About 50% (8/17) of the countries have population sizes of less than 10 million. The mean proportion of people over 60 years is 6%, which contrasts sharply with the global average of 12%, thus indicating a generally younger population. The corresponding figures in the HICs of the group are less than 5%, while the global average for HICs is 22% indicating the youthfulness of the population in the region. In 12/17 of the countries, the annual rate of natural increase of the population is less than 2%. However, the annual growth of the population (inclusive of population migration) is worth noting in the HICs of the group, particularly Qatar and the United Arab Emirates, where the figures are 11.9% and 10.2% respectively. There is a net inflow of people from other parts of the world and it will be important to consider this demographic dynamic in the roadmap towards UHC.

### Health status and risk factors

#### Life expectancy and mortality

The overall health situation in the region has improved since 1990, with improvements in under-5 mortality rate, maternal mortality ratios, and other health indicators. In 2013, the average life expectancy at birth in the region was 74 years, ranging from 63 years in Sudan to 80 years in Lebanon. The mean life expectancy at birth in the HICs of the group is 8 years more than that of the LMICs in the region. Over a period of 23 years (1990–2013), life expectancy increased by an average of 6 years, with a range of 1 year (Iraq) to 13 years (Lebanon). Moreover, the overall mean healthy life expectancy is 10 years less than the unadjusted life expectancy.

**Fig. 1:**
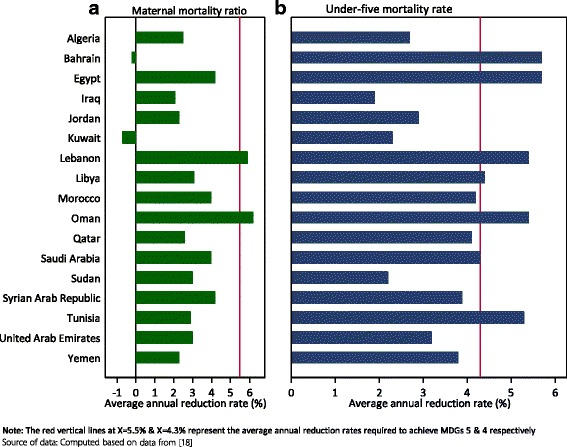
Annual average rates of reduction – Maternal mortality ratio and under-five mortality rate by country

Under-5 mortality has declined significantly in the region since 1990. However, in the period 1990–2013, only Bahrain, Egypt, Lebanon, Oman and Tunisia registered an annual average reduction rate (AARR) of over 4.3% - a rate that is required to achieve the MDG 4 target of reducing under-5 mortality rate by two-thirds between 1990 and 2015. Hence, the majority of the countries (12/17) lag behind and are unlikely to have achieved the targets in the remaining 2 years. Figure 1 presents the average annual average rates of reduction for under-5 mortality rates and maternal mortality ratios for the study countries.

As can be observed from Fig. 1, only Lebanon and Oman reached an AARR of 5.5% required to achieve the MDG 5 target of reducing the maternal mortality ratio (MMR) by three-quarters, between 1990 and 2015. Counterintuitively, in two high-income countries (Bahrain and Kuwait), an increase in MMR was witnessed in the period 1990–2013.

#### Burden of disease

Non-communicable diseases (NCDs) and injuries account for more than 75% of total disability-adjusted life years (DALYs) in the region except in the two lower middle-income countries, Sudan and Yemen, where they account for less than half of the burden of disease. Infectious and parasitic diseases also account for a significant share of DALYs in the latter two countries. DALYs quantify the number of years of life lost due to both premature death and disability. One DALY represents 1 year of healthy life lost.

**Fig. 2:**
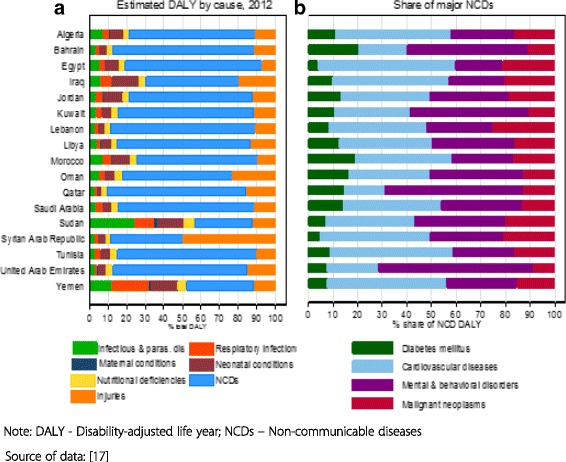
Burden of disease

Cardiovascular disorders (CVDs), mental and behavioral disorders, diabetes mellitus and malignant neoplasms constitute over 60% of the NCDs disease burden in most of the countries. A point worth noting is that mental and behavioral disorders comprise a significant share of the DALYs. This is more pronounced in the HICs in the group, where they pose a greater burden than that of CVDs, diabetes mellitus and malignant neoplasms (Fig. 2).

The mean age standardized DALY rate per 100,000 population is 33,102. However, the rate exhibits marked disparity between LMICs and HICs in the group – the rate being almost twice in the LMICs as compared with that of the HICs (46,295 vs. 25,187).

#### Risk factors

**Fig. 3:**
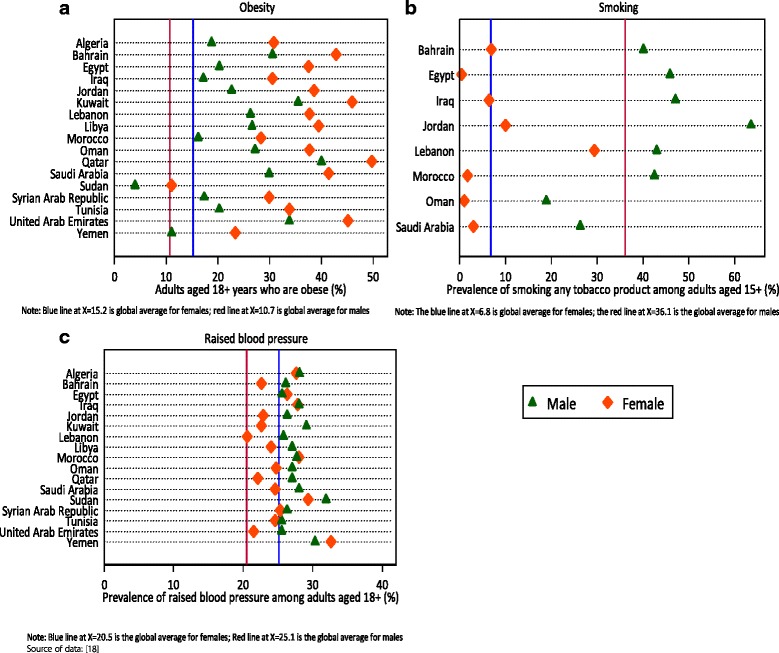
Prevalence of risk factors – smoking and obesity in adults

The prevalence of some risk factors for non-communicable diseases is far higher than global averages (Fig. 3). The mean prevalence of raised fasting blood glucose in the population aged 18 years and above is 16.2% for males and 15.9% for females. These rates are almost two-fold the global average values and pose great concern. In each of the countries considered (17/17), the rate of raised blood glucose level is higher than global averages for both males and females. Similarly, the mean rates of raised blood pressure for males and females respectively are 27.4% and 25.1%, and are still higher than global averages by 2 to 5 percentage points.

In all 17 countries, the obesity rate among females is higher than that of males (Fig. 3). Furthermore, in all HICs in the region, obesity rates among females are over 40% and higher than those of their male counterparts (mean of 35.5% vs 23.4%). These rates compare unfavorably with the global average obesity rate of 15.2% among females. The rate is also more than twice in males when compared to the global average rate of obesity (23.4% vs 10.7%). Out of 8 countries that have data on the prevalence of smoking and tobacco product use among individuals aged 15 years and above, 6 countries have rates above 40% in males (as compared with a global average of 36.1%). However, only three countries have rates in females exceeding the global average of 6.8%. Two countries are found to be outliers: in Jordan, the rate is 63.6% among males, and in Lebanon it is 29.4% among females, thus raising extra concerns.

### Health systems

#### Health workforce

The mean physician density in the region (20 physicians per 10,000 population) is higher than the global physician density of 13.9. The same is true for the nursing and midwifery personnel density (36.8 vs. 28.6 per 10,000 population). However, comparison with averages of countries with similar income status reveals that physician density in many countries of the region is lower. Out of the six high-income countries in the group, all but Qatar have physician densities that are lower than the HICs global average of 28.7 physicians per 10,000 population for the period 2007–2013. Similarly, of the six upper middle-income countries, only three have physician densities higher than the global average for upper middle-income countries, which is 16.1 per 10,000 population (Jordan, Lebanon and Libya). Only two lower middle-income countries (Egypt and Syrian Arab Republic) have physician densities higher than the average density for LMICs, which is 7.9. With respect to density of nursing and midwifery personnel, a similar situation is observed.

#### Health expenditures

##### Per capita total expenditure on health

Per capita total health expenditure increased from PPP Int. $797 in 2000 to PPP Int. $1227 in 2014 with a wide variation between the different income groupings. Table 2 and Table 3 present respectively the per capita total expenditure on health for individual countries for 2000 and 2014 and the mean health financing indicators by income group for the MENA region for 2000 and 2014. The mean per capita total expenditure on health for the high-income countries in the group was PPP Int. $ 2330 in 2014, which is more than six-fold that of the LMICs and about 3 times that of the UMICs (Table 3). In 2012, per capita total expenditure on health in the high-income group of countries ranged from less than a quarter to about 60% of the global average for high-income countries, which was PPP Int. $4516. All six UMIC countries spent less than the global average of PPP Int. $766 for a similar group of countries (Table 3).

**Table 2:** Annual per capita total health expenditure by country in International Dollars (purchasing power parity)

Country	2000	2014	AAGR^a^ 2000–2014
Algeria	278	932	9.0
Bahrain	1256	2273	4.3
Egypt	325	594	4.4
Iraq	67^b^	667	17.8
Jordan	593	798	2.1
Kuwait	1478	2320	3.3
Lebanon	1061	987	−0.5
Libya	699	806	1.0
Morocco	147	447	8.3
Oman	1081	1442	2.1
Qatar	1929	3071	3.4
Saudi Arabia	1186	2466	5.4
Sudan	75	282	9.9
Syrian Arab Republic	157	376	6.4
Tunisia	314	785	6.8
United Arab Emirates	2034	2405	1.2
Yemen	146	202	2.4

Source of data: [16]

^a^ *AAGR* average annual growth rate (%)

^b^data is for 2003

**Table 3:** Mean health financing indicator values for the Middle East and North Africa Region by World Bank income group, 2000–2014

Indicator	LMICs^a^		UMICs^b^		HICs^c^	
	2000	2014	2000	2014	2000	2014
Total expenditure on health per capita (US$)	46	129	264	391	814	1311
Total expenditure on health per capita (Int$)	170	380	589	829	1494	2330
Total expenditure on health as a share of GDP	4.4	5.8	6.6	6.4	3.0	3.7
General government expenditure on health as a share of GDP	1.7	1.7	3.0	4.1	2.2	2.8
General government expenditure on health as a share of general government expenditure	7.1	6.4	9.6	10.0	7.3	7.6
General government expenditure on health as a share of total expenditure on health	38.2	32.4	51.0	63.7	73.1	78.7
Out-of-pocket expenditure as a share of total expenditure on health	56.1	63.9	41.8	31.2	20.7	13.5

Source: computed from [16]

^a^ *LMIC* lower middle income country

^b^ *UMIC* upper middle income country

^c^ *HIC* high income country

##### Total expenditure on health as a share of gross domestic product (GDP)

The health system constitutes a small part of the economies of most countries in the region. The GDP share of total expenditure on health increased from 4.5% in 2000 to 5.3% in 2014. The figure was the lowest for the HICs in the group: 3.7% in 2014. In 2012, the share of total expenditure on health in the region was 5%, which is much lower than the global average of 8.6%. In the same period, the figure for HICs of the group was 3.2% as compared with a global average of 11.6% for high-income countries.

##### General government expenditure on health (GGHE) as a share of GDP

**Fig. 4:**
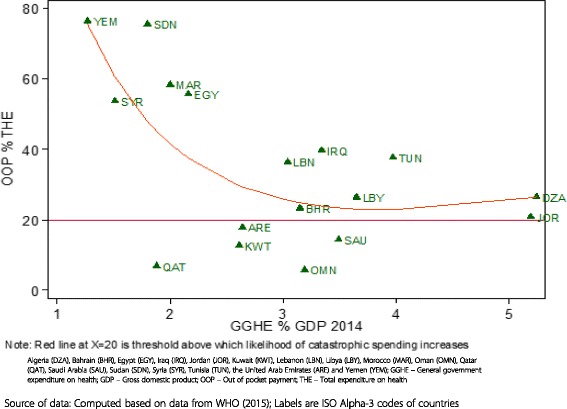
General government expenditure on health as a percentage of gross domestic product vs. out of pocket spending as a percentage of total health expenditure, 2014

The GGHE as a share of GDP, increased from 2.4% in 2000 to 2.9% in 2014. The upper middle-income countries had the highest figure of this indicator in 2014 (4%), while those of the lower middle income and high-income ones were 1.8% and 2.8% respectively in 2014. The GGHE as a share of GDP is relatively low in the high-income countries. However, it is observed not to be associated with high out-of-pocket spending that exceeds the threshold of 20% of total expenditure on health above which the likelihood of catastrophic spending increases (Fig. 4).

As can be observed from Fig. 4, although the GGHE % of GDP in the high income countries of the group is less than 3%, the corresponding OOP share of THE is less than 20% in 5 out of the 6 countries. This signifies the need to consider the strength of the economy as measured by the per capita GDP besides the GGHE share of GDP.

##### General government expenditure on health (GGHE) as a share of general government expenditure (GGE)

In the period 2000–2014, the GGHE constituted from 7 to 8% of general government expenditure on average. In 2012, the figure for the group of countries was 8.3% as opposed to a global average of 14.1%. When disaggregated by country income group, the LMICs and UMICs GGHE share of the GGE was in line with global averages for the respective economic group. However, the GGHE as percentage of GGE in the high-income countries in the group was less than half of the global average for high-income countries (7.1% vs 16.8%).

##### General government expenditure on health as a share of total expenditure on health

**Fig. 5:**
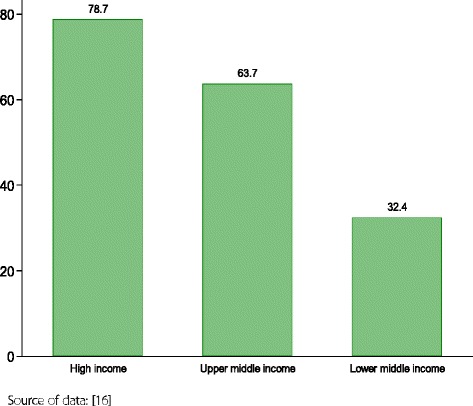
General government expenditure on health as a percentage of total health expenditure, 2014, by income group

The general government expenditure on health increased from 55% in 2000 to 60% of the total expenditure on health in 2014. However, there is a wide variation by country economic groupings as depicted in Fig. 5.

##### Out-of-pocket expenditure (OOPS) as a share of total expenditure on health

**Fig. 6:**
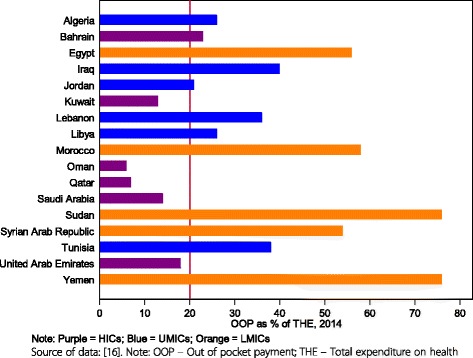
Out of pocket payment as a percentage of total health expenditure, 2014, by country

OOPS as a share of total expenditure on health decreased slightly from 38% in 2000 to 35% in 2013. In 2014, OOPS in the LMICs, UMICs and HICs in the group was 64%, 31% and 13% respectively. The OOPS share of THE is notable in the LMICs. In two LMICs, Sudan and Yemen, OOPS is as high as 76% as shown in Fig. 6.

## Discussion

Results from this study suggest that a range of unique sets of challenges have to be overcome to pave the way toward achieving universal health coverage. Suggested reforms range from public health policies aiming to reduce NCDs, injuries, and their risk factors to policies addressing shortages in human resources, to financing policy reforms including expanding fiscal space, increasing government share of spending on health, and reducing OOPS. The countries have a relatively youthful population, partly attributed to the influx of migrant labor, particularly in the high income group of countries. The health needs of the young segment of the population should therefore be considered in planning health services.

### Burden of disease

The study results reveal worryingly heavy burden of disease from CVDs, mental and behavioral disorders, diabetes mellitus, and malignant neoplasms. The countries are at different stages of the demographic, epidemiological, nutritional and risk factor transitions. The health systems of the LMICs, in particular, face the challenges of both infectious and parasitic diseases and chronic non-communicable diseases, posing further pressure on the relatively meager health system resources available. Knowledge of the stage of health transition of a country is necessary for priority setting and evaluation of health programs [1]. Hence, health policies, strategies and plans in these countries must duly consider the state of health transition of each country to achieve better value for money.

Similar to previous research [20–23], this study shows high obesity rates in the MENA region, which has major implications on the public health systems in the region and the high burden of NCDs. Among adults, the prevalence of obesity ranged from 40% for females to 23.4% for males. National plans should be developed to reduce the health burden of obesity in the region. Recommended policies should address the level of inactivity in the society, the urbanization effects, and the excessive marketing, promotion, and consumption of unhealthy foods [21, 23–25].

To address NCDs and their shared risk factors (obesity, tobacco use, harmful use of alcohol, and physical inactivity), policy makers should strengthen health systems through a people-centered primary healthcare policy and adopt a multi-sectoral, population-wide public health policy reform [24].

The Global Action Plan for the Prevention and Control of Non-communicable Diseases 2013–2020 suggests the use of evidence-based fiscal policies appropriate to the national context. These include taxes and subsidies, to improve access to healthy dietary choices and create incentives for behaviors associated with improved health outcomes and discourage the consumption of less healthy options [24]. Thus, health legislatures should adopt national policies to reduce population salt/sodium intake, limit saturated fatty acids, and eliminate industrially produced trans-fatty acids [25, 26]. In addition, countries are urged to adopt the International Code of Marketing of Breast-milk Substitutes, and the WHO set of recommendations for marketing of unhealthy foods and sugary non-alcoholic beverages to children and within schools [25–27]. For example, imposing taxes on sugar-sweetened beverages especially if these taxes aim on increasing the retail price of beverages by 20% or more was found to lead to reductions in the consumption of these beverages [27]. To curb the high prevalence of tobacco use in the region, countries should accelerate implementation of the WHO Framework Convention on Tobacco Control (FCTC) [25, 28]. The FCTC encourages countries to develop and implement demand-reduction measures such as, (1) imposing tax increases on tobacco products; (2) endorse by law the creation of completely smoke-free environments in all indoor workplaces, public places and public transport; (3) ban all forms of tobacco advertising; and (4) promote through mass media campaigns the dangers of tobacco and smoking [28].

### Expanding fiscal space

Fiscal space for health is the availability of resources or budgetary room to increase a government spending on health without jeopardizing the stability of the economy or affecting other sectors [29]. Even if a government wants to increase public spending on health, the lack of fiscal space may limit this from happening [29, 30].

The results reveal that the HICs and some of the UMICs in the group have seen an increase in per capita total expenditure on health and a decreased share of OOPS, manifesting signs of health financing transition [31]. This implies that the road to UHC in the LMICs and some of the UMICs will require enhanced political commitment and generation of more resources earmarked for health. Increasing health spending by giving more priority to health is one of the key determinants of fiscal space directly under the control of government decision that LMICs should consider [32]. Furthermore, improving the efficiency of health spending is also an area that all income groups of countries should explore. The World Health Report 2010 estimates that globally 20–40% of health resources are wasted [7]. A key policy area to realize efficiency savings would be enhancing the purchasing function of health financing [32].

The region witnessed a progressive decline in mean (GGHE) as percentage of (GGE) during the period between year 2000 and 2008. After that period, the mean gradually rose to its levels in the period pre-year 2002. Fiscal space should have automatically increased as the countries’ economies grew especially with the rise in GDP for HICs in the group. However, the GGHE as a share of GDP in HICs remained relatively low.

Giving higher priority for health in governments’ budget allocations should lead to a greater fiscal space [30, 33]. Countries in the MENA region varied in their public share of spending on health. The mean GGHE as a share of THE varied from as low as 32.7% for LMICs, to 63.7% for UMICs, to 78.7% for HICs. When the proportion of total health expenditure coming from public sources falls below around 70–80%, which is the case for LMICs and UMICs in this group, research suggests that the number of households falling into financial difficulties increases significantly [33, 34]. Increasing public spending for health care depends on the fiscal capacity of government, which is proxied by general government expenditure as a percentage of the GDP and the share of GGHE in general government expenditure [35]. A rule of thumb suggests that government spending in excess of 35% indicates high fiscal capacity, where as a figure less than 20% is indicative of low fiscal capacity [36]. In the LMICs of the group, notably Sudan and Yemen, the fiscal capacity is low (12.9% and 23.5% respectively in 2015) [37]. Thus even if this is coupled with a very high government commitment to health care, public spending on health will remain low exposing individuals and households to financial hardships. This calls for the introduction of innovative financing mechanisms to supplement the public spending on health and increased development assistance for health to these countries.

To move toward UHC, financial policy reform in the MENA region should aim at increasing public revenues for the health sector. This could be achieved by first increasing the priority given to health in governmental budget allocations despite current fiscal constraints [29, 33, 38]. Second, improving tax administration and raising new taxes will help grow the fiscal space for health [30, 38]. This could be achieved by the introduction of sin taxes; earmarked consumption taxes; extension of taxation base; and value-added tax [38]. Finally, although external funding is an important source of public funding for health especially in LMICs and UMICs as they are receiving huge numbers of refugees due to conflicts in region, its unpredictability and inflexible nature [39] may likely pose challenges to the health systems of these countries. To address these challenges, the international community has to ensure predictability of external health resource flows to the LMICs and UMICs that host a large number of refugees in the region.

### Reducing out of pocket spending

Financial protection in health is achieved when payments made by people towards the cost of using health services do not severely impact their living standards [40, 41]. Financial protection is commonly measured in terms of “catastrophic” and “impoverishing” payments resulting from OOPS [40–43]. Catastrophic payments capture whether out-of-pocket payments exceed some threshold of household income [41]. In this case, the OOPS are so high compared with the household income which may push households to give up other essential goods and services. The impoverishment measure captures whether households fall into poverty through out-of-pocket spending [41]. Impoverishing OOP payments may push a household below, or further below, the poverty line. According to the WHO, financial catastrophe is when direct out-of-pocket payment exceeds 40% of household income net of subsistence needs [44].

However, in this study and due to lack of household expenditure surveys in some countries in the MENA region and publically available data, the ratio of out of pocket spending to total health expenditure (OOPs/THE) is used as indicator of financial protection. This is mainly because of the high correlation between this indicator with the incidence of financial catastrophe (and impoverishment) [44]. The study results show that most countries in the MENA region depend on OOPS. OOPS ranged from as low as 6% in Oman to 76% in Sudan and Yemen in 2014. The GCC countries spent less out-of-pocket on health services than the remaining MENA countries. In most countries except for Kuwait, Oman, Qatar, Saudi Arabia, and the United Arab Emirates, OOPS exceeds 20% of total health spending which is a threshold below which the risk of catastrophic spending is generally small [35]. Hence, there is a high risk of catastrophic spending in the MENA region. The highest risk is observed in the LMI group of countries.

High OOPS results in people seeking less healthcare treatment, skipping treatment, or not seeking care at all specially for those countries in the MENA region with public spending on health less than 70 to 80% of total health spending [31, 33].

To reduce OOPS, policy-makers will need to develop different approaches to (1) extend population coverage through prepayment mechanisms; (2) protect the poor and disadvantaged; (3) design a benefits package; and (4) decide on the appropriate level of cost sharing by the patients [45, 46].

### Addressing shortages in human resources

The MENA region will face difficulties in achieving SDG 3c by 2030, which aims to “Substantially increase health financing and the recruitment, development, training and retention of the health workforce in developing countries, etc. [3]” First, physicians and nursing density in many countries of the region were lower than global averages when compared with countries with similar income status. This could be an indication of lack of policy to strengthen health workforce in the region [47], inability to generate sufficient economic demand to meet current healthcare needs in LMICs, or a rapid growing demand for healthcare workforce (with the rise in NCDs) and shortages in underserved and remote areas in UMICs and HICs in the group. Second, the study results reveal a decline in physician densities in several countries in the region. This could be an indication of significant challenges in the region in recruiting and retaining health workforce, and the limited availability of medical schools and medical training opportunities. In the Gulf Cooperation Council (GCC) countries for example, the evidence suggests that about 80% of medical staff are from outside the region and have received training in more than 50 different countries [48]. GCC countries are starting to focus on encouraging their nationals to receive training and take roles in the healthcare sector, while creating educational frameworks to ensure the development of their skills [49]. Despite high-profile collaborations with international medical schools, the numbers of new medical graduates is not likely to keep pace with the expected population increase. At the same time, while LMI countries such as Sudan have low rates of physicians to start with, the majority of their healthcare workforce are moving to work in GCC countries [50].

## Conclusion

Our study reveals a number of systemic problems in the healthcare systems of MENA countries. The high rates of non-communicable diseases in most countries is a sign of changing lifestyles and eating habits that are having increasingly detrimental effects on health. While most countries’ economies have grown in the last period, governmental spending on healthcare services have not kept pace, thus revealing that healthcare is still not considered a top national priority. As a result, people are spending more and more out of their own pockets to receive healthcare services, which reduces access to needed care and limits funds available for other life necessities. These health and risk factor transitions have profound effects on the quantity and type of health services required in the region [51]. Health policies, strategies and plans have to consider these phenomena in order to implement evidence-based cost-effective interventions to achieve universal health coverage and other targets of the SDGs.

## Abbreviations

AARR: Annual average reduction rate
CVD: Cardiovascular disorder
DALY: Disability-adjusted life year
FCTC: Framework Convention on Tobacco Control
GDP: Gross domestic product
GGE: General government expenditure
GGHE: General government expenditure on health
GNI: Gross national income
HDI: Human development index
HIC: High-income country
LMIC: Lower middle-income country
MDG: Millennium Development Goal
MENA: Middle East and North Africa
MMR: Maternal mortality ratio
NCD: Non-communicable disease
OOPS: Out-of-pocket spending
PTSD: Post-traumatic stress syndrome
SDG: Sustainable Development Goal
THE: Total health expenditure
UHC: Universal Health Coverage
UMIC: Upper middle-income country
UNHCR: United Nations High Commissioner for Refugees
WHO: World Health Organization
